# Ultrasound Powered Implants: Design, Performance Considerations and Simulation Results

**DOI:** 10.1038/s41598-020-63097-2

**Published:** 2020-04-16

**Authors:** Bruno Miguel Gil Rosa, Guang-Zhong Yang

**Affiliations:** 1 The Hamlyn Centre, Imperial College London, London, SW7 2AZ UK; 2 Institute of Medical Robotics, Shanghai Jiao Tong University, Shanghai, 200240 China

**Keywords:** Computational models, Biomedical engineering

## Abstract

Ultrasounds (US) has been used in the past decades as a non-invasive imaging modality. Although employed extensively in clinical applications for soft tissue imaging, the acoustic beams can also be used for sensing and actuation for biological implants. In this paper we present a unified three dimensional (3D) computational framework to simulate the performance and response of deeply implanted devices to US stimulation and composed by a double piezoelectric layer with different material composition and configurations. The model combines the temporally-invariant distribution of the scattered pressure field arising from the presence of scatterers and attenuators in the domain of simulation, with the time-delay propagation of waves caused by refraction, to solve the Forward Problem in US within the breast and lower abdominal regions. It was found that a lens-shaped implant produces higher peak echoes in the breast for frequencies ≤ 6 MHz whereas, in the liver, similar strengths are obtained for the lens and disk-shaped implants in the higher spectrum. Regarding material composition, a combination of LiNbO_3_ with PZT-5A yielded higher amplitude signals, when the double layer thickness is comparable to the wavelength of excitation. Experimental validation of the proposed model was carried out in the presence of a synthetic anatomical phantom of the breast and water tank to investigate the acoustic signals generated by disk-shaped implants when stimulated by external US sources in the harmonic and impulsive regimes of wave propagation. The implantation of a double piezoelectric layer inside the human body can, in the future, provide a high resolution system for the detection of surgical site infection as well as tumour growth and other systemic inflammatory responses originating deeply in soft tissues.

## Introduction

Ultrasound imaging is a widely used diagnostic and therapeutic tool for a range of clinical applications including, for example, prenatal care, urology and gynaecology, as well as breast cancer screening and assessment of hepatobillary abnormalities^1^. In medical imaging, the sound waves are employed passively to interact with the human body and record the differences in the acoustic properties of tissues as convoyed by the backscattered echoes. However, the exploration of other types of interaction in which the acoustic beam can effectively activate or set into motion a series of physiological events has the potential to provide new clinical applications, such as remote release of chemical compounds in target tissues^2,3^, targeted neuromodulation^4^ and enhancement of optical access to brain activity by photoacoustic microscopy^5^ or cardiovascular pressure monitoring from multiple body locations^6^. The acoustic sound waves can likewise deliver power and telemetry capabilities to deeply implanted devices for remote sensing of the physiological environment in soft biological tissues, as an alternative to inductive (near field) and radio-frequency (RF) links^7^. Advantages of US include immunity to electromagnetic interferences within the physiological environment and the apodization of the acoustic beam that can counterbalance some geometrical misalignments between the internal and external piezoelectric transducers, which has no precedent in both magnetic and RF links, since coils dramatically decrease performance when shifted from the transmission path^8^ and antennas cannot cope with the differences in the dielectric permittivity values found between body tissues and air. Moreover, for the operational frequencies typically employed in US, the penetration depth in tissue is a function of frequency, a fact that can be explored to track the location of the implant itself and the knowledge of the distribution map for the acoustic pressure over the radiated area can be used to maximize power transfer within safe biological limits^9^ (≤720 mW/cm^2^).

The equations governing the interaction between ultrasounds and materials have long been described in terms of pressure and/or velocity, though a closed-form solution is hardly attainable without any mathematical simplification. Several factors are responsible for this behaviour: the refraction, scattering and attenuation of sound waves are both space and time-dependent on many physical parameters of the transmission path, which begin as soon as the rays leave the source. Indeed, the geometry of the transmitting transducer (or array) can be exquisite, preventing a simpler description of the incident acoustic field that does not rely on the solution of an high-order Bessel equation, even for the harmonic regime of excitation^10^. In addition to the cumbersome mathematical formulation, the availability of a computational framework to test the acoustic response of a deeply implanted device in terms of composing materials, dimensions and final packaging is of paramount importance to guide the design process of the device itself^11–13^. Any of these variables can affect the performance at the implant side, specially when moving from bulky electronic components to highly integrated and miniaturised devices. The incorporation of acoustic sensing layers and actuators with signal processing circuits on piezoelectric substrates create systems with improved spatial resolution to detect changes in viscosity, temperature, pH and mass loading, by measuring the variations on the resonant frequency of the substrate, velocity and/or time-delay of sound propagation. The technology of *passive* device interrogation already exists in the form of film bulk acoustic (FBAR)^14^ and surface wave resonators (SAW)^15–17^, but a complete 3D simulation framework including material testing, geometry assessment and evaluation within a biological phantom is still lacking and much desirable in biomedical applications related to energy harvesting and data transmission for implantable devices, bringing these solutions closer to “zero-power” functionality as opposed to their electronically-*activated* counterparts^18^.

In terms of the piezoelectric element or transducer, the different electromechanical coefficients are fundamental to the selection of the ideal ultrasonic-to-voltage converter to be included as the powering unit inside the implantable device. A good combination between the mechanical (robustness) and electrical (voltage) properties allows designing high energy density piezos that can be deployed in constrained spaces inside the human body. The type of materials commonly employed in US encompasses lead zirconate titanate (PZT) and derivatives (PZT-5A or PZT-5H), barium titanate (BaTiO_3_), lithium niobate (LiNbO_3_), polyvinylidene fluoride (PVDF) and co-polymers, aluminium nitride (AIN) and zinc niobate (ZnO). Due to both toxicity and bio-compatibility issues only BaTiO_3_, AIN and ZnO can be certified for medical implantation. However, the electromechanical coupling achieved by these materials is lower when compared to PZT, forcing the deposition of additional bio-compatible layers, like polydimethylsiloxane (PDMS), over PZT or another type of surface treatment to interface biological tissues. The piezoelectric element and acoustically matching layers can be fabricated directly by MEMS processes^19–21^ followed by post-processing techniques involving metal deposition (electrodes), etching, sputtering or lithography, in order to minimize acoustic impedance mismatches between the fabricated transducer and body tissues. Moreover, the fabrication of hybrid energy generators by combining piezos with ferromagnetic^22^ or triboelectric^23^ materials has also been shown to increase the harvesting power, in addition to offer the possibility of external activation by magnetic fields or mechanical loading.

Medical applications that can benefit from a reliable implantable device activated by US include the monitoring of the physiological and pathological condition of soft tissue not completely encircled by bone. Detection of tumor growth in its early stages may not be accomplished using the traditional MRI, CT and PET scans, neither for other systemic inflammatory responses originating deeply in the interstitial body parts^24,25^. Also, the detection of tissue infection originating after surgery is of clinical relevance: the piezo layers can be designed to fill a patch area or the stitches that surround the surgical site and left there for the time necessary to assure proper healing while revealing physical parameters of the tissue after passive interrogation by an ultrasonic scanning device. The concept of ultrasonic trancutaneous energy transfer (UTET) was originally proposed by Rosen *et al* (1958) and attempts to produce acoustically-activated medical implants have been made by Ozeri and Schmilovitz^7^ with a subcutaneous power delivery system using either uniform or non-uniform wave excitation for devices located 40 mm deep in a water tank; Sanni *et al*.^26^ used an inductive (subcutaneous) and ultrasonic (soft tissue) multi-tier interface to access analogue sensors deployed 70 mm deep in agar-filled solution, with harvesting power levels of 29 *μ*W; again, Ozeri and Schmilovitz^10^ proposed a single PZT for both power harvesting and backward data transmission, using small variations (≤10%) of the electric circuit load directly connected to the PZT and achieving data transfer rates of 1200 bps inside a water tank; Charthad *et al*.^27^ with a hybrid bi-directional data communication link (UWB RF) powered by US have achieved a level ≥ 100 *μ*W for a 4 mm × 7.8 mm *system-on-chip* embedded on chicken meat at depths of 3 cm; Shi *et al*.^28^ presented an MEMS-based PZT harvester with wide operational bandwidth by combining 7 PZT diaphragms (dimensions of 500 *μ*m × 250 *μ*m each) in parallel in order to increase output power, which enabled the harvester to achieve a performance boost by a factor of 6 for the power output density (0.59 to 3.75 *μ*W/cm^2^) simply by changing the transmission frequency from 250 to 240 kHz, at distances source-harvester of 1 cm; by its turn, Seo *et al*.^29^ reported an ultrasonic neural dust system (volume of 2.4 mm^3^) for powering and communication purposes with experimental validation *in vivo* by recording the electroneurogram and electromyogram from the rat peripheral nervous system and skeletal muscle; Lee *et al*.^30^ developed a MEMS ultrasonic transducer composed of 16 elements made of PZT for generation of electric power and stimulation of neuron cells in culture; finally, in a complete different application, Kim *et al*.^31^ developed and tested in mice an US-powered implantable device (720 kHz) with embedded light sources to deliver *in situ* photo-dynamic therapy to deep-seated tumors, achieving between 0.048 to 6.5 mW/cm^2^ of optical power from transducers made of PZT-5A with total volume ≤16 mm^3^. Nonetheless, the level of harvested power achieved by previous devices from UTET is still too low to fulfill the requirements of reliable sensing or actuation in the physiological environment as achieved by modern battery-powered pacemakers and neurological stimulators (≥100 *μ*W)^32^, barely operating afloat of their limited specifications. Challenges posed by the physiological environment (saline medium) and geometry of body tissues to which the implantable device attach divert the acoustic beams from homogeneous propagation and need to be properly characterized by simulation models mimicking the exact anatomical structures within the transmission path before real deployment inside the body.

It is the objective of this paper to provide a 3D computational framework with a multi-level grid resolution to solve the spatial and temporal Forward Problems in Ultrasounds for remotely-implanted devices in sectional areas of the human body - breast and abdomen (liver) - by means of a two-step approach running in parallel: the computation of a domain-sized *backscattering operator* and a *ray-tracing operator* for the individual acoustic rays. To that end, the complete derivation of the mathematical relations governing acoustic wave propagation will be presented, separating the effects in space (scattering, attenuation) from those related to time-delay propagation (refraction). Since the calculation of the scattering operator involves mapping each point (or element) in the domain to the remaining ones, it has complexity ${\mathscr{O}}$(*N*^2^) limiting the number of simulation points, which may cause interface discontinuities within the domain to be imperfectly discretised. However, a single solution of the operator yields a full-domain *scattered* map as if the rays emanating from the source reach every location with exact the same time, resembling a *static* field distribution derived from the *near-field* regime of propagation. The ray-tracing operator introduces directional and phase-delay variations in the acoustic beam with a *time-of-flight* dictated by the computational overload imposed by the model. In this *far-field* scenario, the number of wavelengths for propagation of the rays will create a higher resolution volume that can be used to model more accurately tissue interfaces.

## Results

### Scattered acoustic field

**Figure 1:**
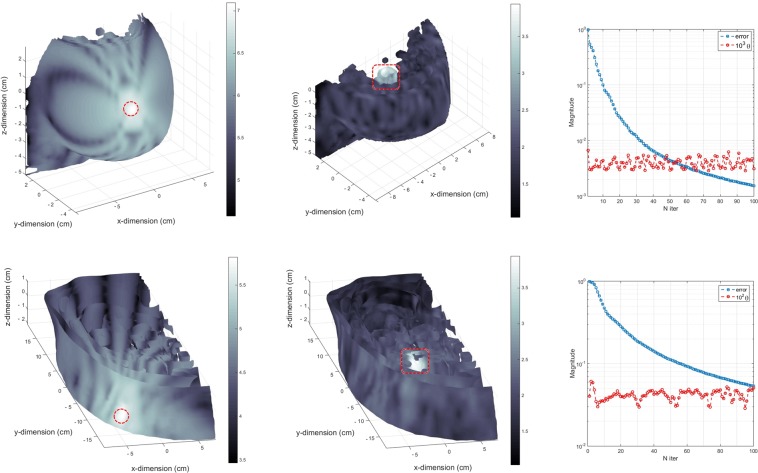
Simulation results and performance of the scattering operator for the breast and lower abdominal phantoms with uniform US excitation (5 MHz). (**a**) Incident pressure field projected onto the walls of the breast with color-bar levels presented in a logarithmic scale. The location of the source transducer is shown by the red dashed circle in the image (frontal view of the anatomical slide). (**b**) Scattered pressure field produced by the lens-shaped implantable device located inside the breast (red dashed rectangle), as given by the solution of the CG routine (transverse mid-sectional cut). (**c**) Evaluation of the error norm and $\theta$ parameter along the iterations of the numerical routine for the breast phantom. (**d**) Incident pressure field projected onto the external walls of the right lumbar region (frontal view). The source transducer is shown by the dashed red circle located in an anterior position, halfway between the mid-line of the body and the mid-axillary line. (**e**) Scattered pressure field obtained by a lens-shaped implant located inside the liver (red dashed rectangle) and facing diagonally the source transducer. (**f**) CG parameters evaluated along the iterations of the numerical routine for the lower abdominal phantom.

Figure 1 shows the pressure field distributions for the breast and lower abdominal phantoms obtained by the resolution of the scattering operator, ${{{\mathscr{O}}}_{{\mathscr{P}}}}^{sct}$. A lens-shaped implant was employed during these simulations with dimensions of 1 cm × 1 cm × 0.5 cm and composed by a double piezo layer made of PZT-5A/LiNbO_3_ (thickness of 1 mm/2 mm). Description about the implementation of the discrete system of equations governing wave propagation can be found on **Methods, Calculation of the scattering operator** and the spatial discretization of the 3D domain and phantom segmentation can be found on sections **Methods, Computational mesh** and **Methods, Phantom segmentation**, respectively. The implantable devices to be deployed inside the breast and liver structures can adopt geometrical configurations in the form of a lens, disk or cone with two piezo layers encompassed by an external layer of bio-compatible PDMS material as further described on section **Methods, Implantable device design**. The simulations are performed inside Matlab (Mathworks Inc., Natick, MA, USA) running on a Intel Core i7-4770 CPU at 3.4 GHz with 16 GB of RAM. The average time required for the iterations are 15 and 20 minutes for the breast and abdominal phantoms, respectively, due to the dimension of the 3D domains and manipulation of complex-valued physical quantities. The incident pressure fields are calculated only once before the numerical routine and so they do not account for the aforementioned iteration time. The computational mesh and topological relations between the elements are also pre-loaded from stored files in memory, since their edification is quite time consuming and do not change during the iterative processes. In the simulations, the criteria for interruption is set at an iteration number of 100 whenever the *error* decreases monotonically (*l2*-norm), with an acceptable error norm below 10%.

### Echo pulses and frequency spectrum

**Figure 2:**
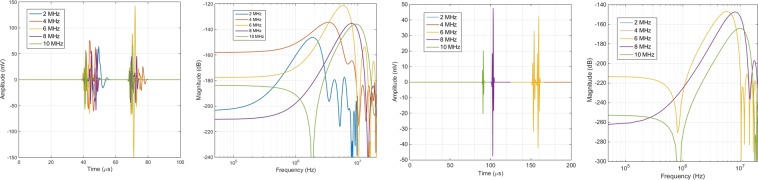
Examples of echo signals picked-up externally for a non-uniform stimulation of the source transducer (Gaussian pulse) with selected frequencies. (**a**) Voltage signals generated by acoustically exciting the breast implant with a lens-shaped configuration whose layers of PZN-PT and LiNbO_3_ are 1 mm and 2 mm thick, respectively. (**b**) Frequency spectrum of the echo signals for the breast implant. (**c**) Echoes produced by a lens-shaped implant deployed inside the liver, with visible absence of signals for the lower frequencies in the time-window provided. (**d**) Frequency content of the signals for the liver implant.

For the ray-tracing operator, represented by ${{{\mathscr{O}}}_{{\mathscr{P}}}}^{ray}$, the transducer is excited by a Gaussian pulse whose mathematical formulation is given by Eq. 1, with amplitude set to 10 V and time duration dictated by the employed frequencies $\in$ $\mathrm{[1,10]}$ MHz. The complete formulation of the operator can be found on section **Methods, Calculation of the ray-tracing operator**. Figure 2 displays the voltage signals or *echoes* detected by a transducer occupying the same position as the source of US, as well as their frequency content, within the assumption of a perfect electromechanical coupling for the transducers in converting a voltage value to a pressure equivalent and *vice versa*. The average time for each echo computation is frequency and spatial-dependent, lasting from an average 5 minutes for the lower frequencies present in the breast phantom to 20 minutes for the higher frequencies in the lower abdominal region.

1 $$v(t\mathrm{)}=10{\rm{s}}{\rm{i}}{\rm{n}}(\omega t){\rm{e}}{\rm{x}}{\rm{p}}\left\{\frac{-{\left[0.2\omega \left(t-\frac{8{\Pi }^{2}}{\omega }\right)\right]}^{2}}{20}\right\}$$

Parameters like the number of pulses detected within the time window allowed for acoustic beam propagation, peak amplitude and convoyed energy will assess the performance of the different implant configuration, composition and layer thickness. The detected pulses are related to the number of acoustic rays reaching back the detection transducer, whose amplitudes are larger than 10 *μ*V in order to limit the computational overload, also referred as meaningful interactions throughout this manuscript. By its turn, the energy convoyed by the echoes is proportional to their squared amplitude calculated for the time-window *T* for US scanning, as given by Eq. 2.

2 $$E=\mathop{\sum }\limits_{t\mathrm{=0}}^{T}|x(t{)|}^{2}$$

### Number of interactions and energy of the acoustic rays

**Figure 3:**
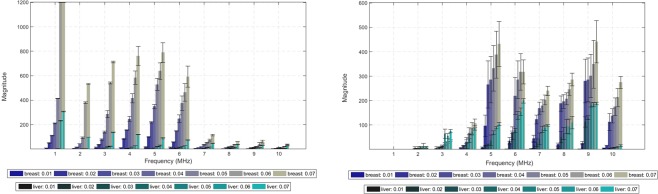
Bar graphic plots (with associated error-bars) for the acoustic rays reaching back the detection transducer with aperture diameter in the range $\in$ [0.01, 0.07] m and averaged along the different configuration and layer composition allowed for the implantable device in the breast and liver tissues (constant thickness of 1 mm/2 mm). (**a)** Number of meaningful interactions detected as a function of the US excitation frequency. (**b)** Energy convoyed by the echo signals by frequency band.

**Figure 4:**
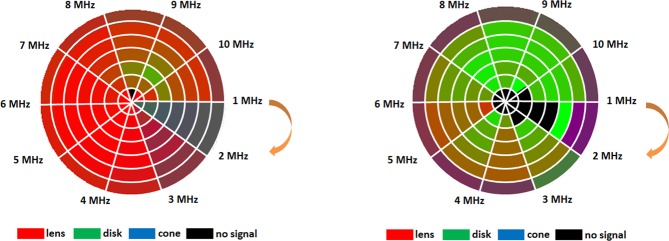
Circular intensity colour plots depicting the variation of the detection diameter (circular) with frequency (line segments). The intensity reflects the contribution of each implant configuration to the overall number of interactions recorded for frequency band. The RGB code of colour is here adopted to give a glimpse of the predominant implant configuration that deviates from the equilibrium (grey colour), when the contribution of each configuration is 0.33. (**a)** Breast phantom. (**b)** Lower abdominal phantom.

Figure 3 displays the number of interactions (and respective energy) as an average value obtained for all the different implant configuration (3 in total) and piezoelectric layers (6 in total). The graphs also present the evaluation for the diameter of the detector or aperture (7 in total $\in$ [0.01, 0.07] m, 0.01 m step) on the same quantities for each frequency band. The limit on the number of interactions was set to a level of 1200 in software in order to prevent further computational calculations since no significant repercussions on the energy level convoyed by lower frequency rays was observed, as Fig. 3b attests. The standard deviation from the average values is also shown in the spectrum for each aperture diameter. In order to spot the influence of each implant configuration on the overall distribution of detectable interactions, Fig. 4 depicts intensity colour graphs for each phantom that reflect the individual contribution of the three implant shapes as a mixture of the RGB code. The white circular lines delimit the diameter for the detection transducer, whereas the line segments spreading radially from the centre bound the frequency bands. The predominance of a particular implant configuration over the spectrum gives an higher tonality to the respective colour, whereas the absence of any dominance is shown as grey and the detection of no signal at all is blacked. From the graphs it is evident a large dominance of the lens-shaped implant in the breast phantom, whereas the disk has more relevance for the liver. The cone shape reveals to have little effect on the overall number of interactions for both phantoms.

### Implant layer composition and thickness

**Figure 5:**
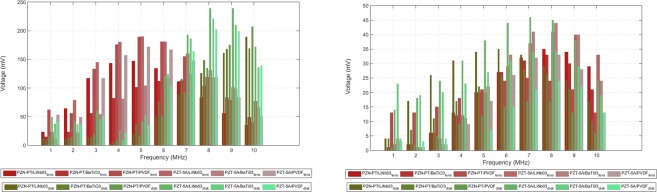
Peak amplitude for the echo signals picked-up externally as a function of the excitation frequency, diameter of detection and composition of the double piezoelectric layer for the implant. Six different double layers are simulated in this paper for the lens and disk-shaped implants only (thickness set to 1 mm/2 mm). Absence of detectable signal for the cone shape predominates for most of extension of the spectrum and so its representation is excluded from the plots. (**a**) Breast phantom. (**b**) Lower abdominal phantom.

**Figure 6:**
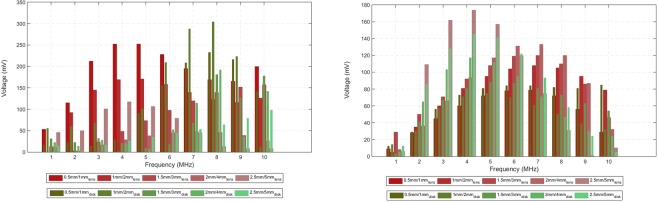
Peak amplitude for the echoes detected by the external transducer as a function of frequency, shape of the implant and thickness of the double piezoelectric layer. The materials composing the double layer are PZT-5A/LiNbO_3_ and their thickness is evaluated by simulation in the lens and disk-shaped implants. (**a**) Breast phantom. (**b**) Lower abdominal phantom.

The evaluation of the double piezoelectric layer composition on the performance of the US transmission line individually for each phantom is shown in Fig. 5, with detection diameter set to 3 cm. The parameter being tested in these graphs is the peak voltage amplitude of the echo signals recorded for the lens and disk-shaped implants only with constant thickness. In general, for a layer thickness of 1 mm/2 mm, the PZT-5A/LiNbO_3_ has achieved a superior performance which makes the combination of these materials suitable for additional testing in order to assess the influence of layer thickness on the implant. Finally, Fig. 6 depicts the influence of layer thickness on the detected echoes in the range $\in$ [0.5, 5] mm.

### Experimental validation of the numerical simulations

**Figure 7:**
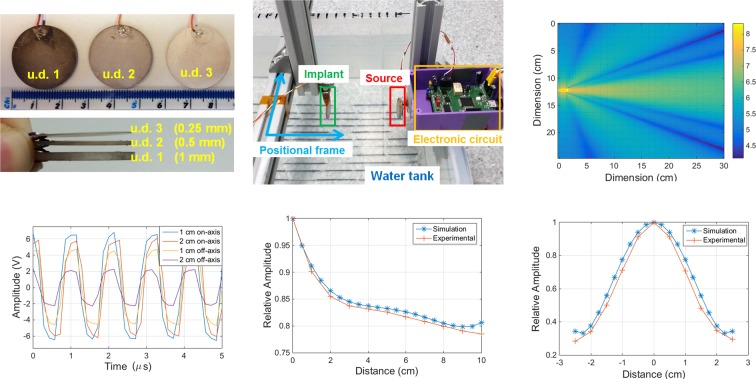
Experimental setup developed for validation of the numerical simulations in a water tank. (**a**) Piezo elements (2.5 cm diameter, single layer of PVDF) used to test the ultrasonic transmission path between a source transducer and implant (receiver) in pairs due to the difference in thicknesses and, therefore, resonant frequencies (u.d. 1: 1 MHz, u.d. 2: 2 MHz and u.d. 3: 4 MHz). (**b**) Water tank (dimensions: 30 cm × 25 cm) containing a fixed source transducer connected to control electronics and a positional metallic frame for changing the location of the implantable transducer relative to the source of US. (**c**) Scattered pressure field distribution (logarithmic scale) for the water tank in the transverse plane containing the alignment axis between source and implant transducers. (**d**) Voltage signals detected by the implantable transducer (u.d. 1) at different distances from the source and stimulated in the harmonic regime with frequency of 1 MHz: on-axis distance corresponds to the gap along the alignment axis of both piezos, whereas off-axis corresponds to the gap in the perpendicular direction from a central point located 1 cm between piezos (on-axis). (**e**) Variation of the amplitude levels detected by the implantable transducer as a function of the on-axis distance to the source, obtained for the simulation (pressure values) and real experimentation (voltage signals): the relative amplitude is expressed as the ration between the level measured by the displaced implantable transducer in relation to the level at zero distance source-implant. (**f**) Relative amplitude level for off-axis displacement from a central point located at 3 cm on-axis between the source transducer, as obtained by numerical simulations and experimentation.

A set of experimental tests were carried out to evaluate the accuracy of the proposed ultrasonic computational framework by using a water tank (homogeneous transmission medium) and a realistic anatomical breast phantom (inhomogeneous medium). Pairs of disk-shaped piezo transducers made of PVDF material were purchased with diameter of 2.5 cm and varying thicknesses ($\in$ [0.25, 1] mm), as shown in Fig. 7a to be used independently as the US source transducer and implantable device. The first set of experiments took place in the water tank depicted in Fig. 7b, with the source transducer fixed to the wall at one extremity of the tank, whereas the implantable device was allowed to move along the transverse plane containing the central acoustic axis of both transducers, which were positioned at the same height. The source transducer could be stimulated by a sinewave (harmonic regime) or Gaussian pulse (impulsive regime) as dictated by the developed electronic circuit for US excitation, with technical details provided on **Methods, Electronic circuit for experimental validation**, along with the impedance curves obtained for the tested transducers with different resonant frequencies due to varying material thicknesses. The pressure field distribution obtained for the tank (Fig. 7c) as well as the voltage signals (Fig. 7d) detected by the implantable device (harmonic regime) were used to calculate a relative amplitude level for on-axis varying distances between the source and implant (geometrical alignment) and off-axis distances perpendicular to a central point located 3 cm straight away from the source, as shown in Fig. 7e,f, respectively, this for *a posteriori* comparison between the levels obtained by simulation and experimentally.

**Figure 8:**
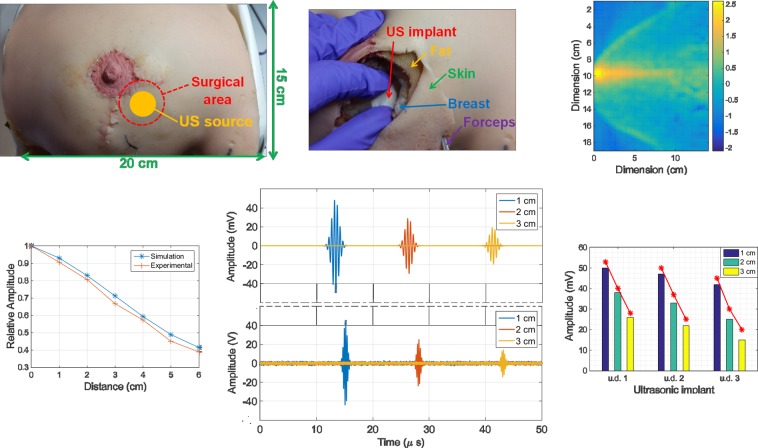
Experimental setup developed for validation of the numerical simulations in the breast model. (**a**) Realistic anatomical phantom used in breast reconstruction surgery training with incision lines for access to the implantable device (transducer) and surface area for contact with the source transducer. (**b**) Insertion of the implantable device (u.d. 3) inside the breast and respective tissue layers (skin, fat, breast) made of materials with similar acoustic properties, namely polydimethylsiloxane (PDMS), silicone and ballistic gel. (**c**) Scattered pressure field distribution (logarithmic scale) in a transverse plane of the phantom at the level of the central acoustic axis from the source transducer. (**d**) Relative amplitude levels as a function of the on-axis distance between the source and implant, obtained from simulation and real experimentation. (**e**) Echoes detected by the source transducer for varying on-axis distances source-implant (1 cm, 2 cm, 3 cm), as given by simulation (top) and real experimentation (bottom). (**f**) Maximum amplitude level of the echoes produced by the different implant types tested (u.d. 1, u.d.2 and u.d. 3), with varying distance to a similar source transducer (1 cm, 2 cm and 3 cm) and represented in a bar plot superimposed by the values obtained by numerical simulation (red lines).

In a similar way, we used the realistic anatomical breast phantom depicted in Fig. 8a to insert the different disk-shaped implantable devices into the breast tissue (Fig. 8b), followed by closure of the surgical opening with tread wire (stitching) so the source transducer could transmit acoustic waves directly over the implant through the surface of the phantom. Figure 8c then shows the scattered pressure field map employed to calculate the relative amplitude level in the harmonic regime of wave propagation in conjunction with the voltage levels detected by the implant, for on-axis distances source-transducer only (Fig. 8d). Finally, the impulsive regime was also tested with the breast phantom, for implants placed at different depths (1 cm, 2 cm and 3 cm) inside the phantom, yielding detection echoes of the same type as presented in Fig. 8e for a 4 MHz stimulation frequency (u.d. 3). By repeating this procedure for all the three available pairs of piezo transducers, the maximum amplitude of the returning echoes can be compared with the equivalent levels estimated by simulation as shown in Fig. 8f.

## Discussion

A computational model to evaluate wave propagation in tissues has been presented that can couple with multiple acoustic interference phenomena by splitting the problem into two separate methodologies in US: the estimation of the backscattering and ray-tracing operators. The computation of the scattering operator suffers from lower image resolution in space but provides time-independent approximations to the distribution of the pressure fields. The ray-tracing operator provides higher spatial resolution but its efficiency depends on the time-step required to sample the US excitation pulse and the limitation on the *time-of-flight* for ray propagation, preventing interactions with tissues located distantly within the domain.

The magnitudes of the scattered fields obtained for both phantoms show a decrease of two orders in magnitude when compared to the incident pressure field (Fig. 1) which validates the assumption that the total acoustic field is only a slightly variation of the incident field and, this way, the Bohr approximation can be used for larger scale simulation domains. In terms of the convergence of the CG routine, the breast phantom has a faster rate when compared to the liver due to the discrepancy in domain size between phantoms (Fig. 1c,f). The error norm continues to decrease monotonically to lower levels at the expense of larger iteration numbers. Another iterative schemes like the Bi-conjugate gradient and nonlinear methods could have been implemented which are characterized by faster convergence rates but, for the size of the 3D US system of equations, a small change in the contrast functions would inevitably result in algorithm instability.

For the ray-tracing operator, the results show that, for the lower frequencies, there is a significant number of detectable interactions with no appreciable energy convoyed by the echo signals (Fig. 3a,b). One of the reasons that can be responsible for this behaviour is the larger wavelength involved in US excitation when compared to the dimensions of the implant, regardless of the shape. With a mean propagation velocity of 4000 m.s^−1^ for the piezo layers, only the central frequencies employed in this study tend to produce enough acoustic beam deflections in the sub-millimetre range. For the breast phantom, the maximum amplitude occurs at the centre of the spectrum in the lens-shaped implant whereas, for the remaining implant configurations, amplitude increases monotonically with frequency (Fig. 2b). By its turn, in the abdominal phantom, all implant configurations lead to a bell-like curve with a steeper decrease towards higher frequencies (Fig. 2d). Since the dimensions of the implantable device are kept the same for both phantoms, this behaviour can be explained by the different distances covered by the acoustic rays, which are larger in the abdominal region, promoting more interactions with the nearby tissues and, eventually, masking the response of the implant itself.

In what concerns implant configuration (constant thickness), the lens-shaped one produces higher peak echoes followed by the disk in the breast phantom, for frequencies up to 6 MHz (Fig. 5a). In fact, for short sensor-detector distances, the semi-circular geometry of the lens provides the most invariant surface to the incoming acoustic rays, by facing directly the source transducer with a 180° angular span. In the opposite direction, a flat surface presents the same normal regardless of the direction of the incident rays and, due to the cone-spreading factor of the beam, the incidence angle decreases off-axis, thus lowering the amplitude of the reflected waves in the lower spectrum. For the liver, the peak amplitudes are more deviated towards the higher frequencies with overall similar strengths for both the lens and disk-shaped implant (Fig. 5b). By its turn, the implant in the form of cone does not produce any echo signal with amplitude comparable to the other configurations (Fig. 4). A cone corresponds precisely to the opposite spatial arrangement of the lens where the rays are forced to follow divergent paths, de-focusing the acoustic beam. Only aperture diameters ≥ 0.07 m can detect these rays and contribute to a more grey-shaded areas in the circular intensity colour plots, although not presented in this paper.

The variation of layer thickness for the lens has shown an increase in signal amplitude when the body of the parabola is comparable to the wavelength of excitation (≤1 mm/2 mm) and so, the employment of thicker layers does not produce any improvement in the detected signal (Fig. 6a). A similar trend is registered for the disk with a slightly increase in signal strength for the higher frequencies as noticed before. However, for the liver implant, the thicker the double layer the higher the magnitude of echoes regardless of the implant shape (Fig. 6b). The exception also occurs for the higher spectrum where some thinner layers predominate in the echoes.

In summary, from all the exposed previously, the lower spectrum of the echoes is highly dependent on the shape of the implant up to a certain implant-detector distance (as seen in the breast), from which layer thickness starts to have a leading role in the amplitude levels of the echoes detected externally (as seen in the liver). For the higher frequencies, changing the configuration and/or layer thickness takes no decisive contribution for the echoes detected in both phantoms.

In terms of material composition for the double layer, a combination of LiNbO_3_ with PZT-5A has produced the strongest signal since the acoustic impedances ($Z=\rho \dot{\nu }$) for these materials are higher than any other combination of the remaining materials, with values of 34.15 MRayls, 33.71 MRayls and 1.5 MRayls recorded for the LiNbO_3_, PZT-5A and biological tissues, respectively (see Table 1, **Methods, Phantom segmentation**). This large mismatch in impedance creates a barrier to the transmission of acoustic rays producing a spot for wave reflection around the implant. In a one-dimensional model of wave propagation at the boundary of the medium, the aforementioned impedances produce a coefficient of reflection of 0.915 and 0.007 in the interfaces tissue/LiNbO_3_ and LiNbO_3_/PZT-5A, respectively. The first interface is, therefore, fundamental for medical applications more concerned in the detection of the reflected signals originated at the implant, rather than assessing what lies beyond as occurs in traditional imaging modalities. The use of more than one piezoelectric layer can be advantageous since different layers can be separately activated by distinctive frequencies and common interferences subtracted from the echo signals produced by the implantable sensor. This constitutes the main reason for considering only numerical simulations with double layers instead of testing them separately.

**Table 1:** Acoustic properties for some biological tissues and piezoelectric materials considered in the present manuscript^1,38,39^.

**Tissue**	*v*	$\rho$	*a*	*y*	**Material**	*v*	$\rho$	*E*	$\eta$	*G*
	(m.s^−1^)	(kg.m^−3^)	dB/MHz^*y*^-cm			(m.s^−1^)	(kg.m^−3^)	(GPa)		(GPa)
Blood	1584	1060	0.14	1.21	PZT-5A	4350	7750	105.8	0.31	40.38
Bone	3198	1990	3.54	0.9	PZT-5H	4560	7500	112.5	0.31	42.94
Breast	1510	1020	0.75	1.5	BaTiO_3_	5470	5700	119	0.32	45.08
Fat	1430	928	0.6	1	LiNbO_3_	7360	4640	156.5	0.35	57.96
Liver	1578	1050	0.45	1.05	PMN-PT	4646	8060	121.5	0.32	46.02
Muscle	1580	1041	0.57	1	PZN-PT	4030	8310	106.8	0.275	41.88
Water	1482	1	2.17e-3	2	PVDF	2200	1780	7.93	0.18	3.36

Note: *E* represents the Young’s modulus, $\eta$ the Poisson’s ratio and *G* the shear modulus.

A limitation of the work presented is that there is a lack of a complete 3D model to represent all tissues with the same edge resolution or the limited time-of-flight allowed for ray propagation. The attenuation phenomena can indeed be responsible for detecting larger signal strengths at higher frequencies. Nonetheless, the results reported in the manuscript show that it is possible to access implanted devices located deeply inside soft tissue and perform modifications to their design, that will have repercussions on the echoes detected externally as a consequence of a reshape in the distribution map of the acoustic pressure field. With a more simplistic geometry for the implantable device (disk-shaped material composed by a single PVDF layer), we were able to match closely the results derived from the simulator with the voltage signals detected by electronics in real experimentation, inside a water tank and anatomical breast phantom. Metrics such as relative amplitude levels produced in the harmonic regime of wave propagation (Figs. 7e,f and 8d), waveform of the echo signals detected and their amplitudes (Fig. 8f) were within acceptable ranges (≤ 10%) below the values estimated by computational simulations, though some deviations in terms of the *time-of-flight* for the echoes detected experimentally were verified (Fig. 8e), along with some measurement noise derived from the experimental conditions themselves (geometrical alignment accuracy and performance electronics).

Finally, in comparison to other commercial software codes available on the market for finite element analysis - such as ANSYS (Canonsburg, PA, USA), COMSOL (Stockholm, Sweden) and OnScale (Redwood City, CA, USA) - the proposed framework for US simulation supplants some of their limitations, by incorporating more computational modules dedicated to automatic image segmentation and labelling from imported CT or MRI scans involved in the generation of the 3D mesh grid, as well as the generation of ultrasonic waves (harmonic and impulsive regimes) and detection by means of interface electronics, specially designed for the type of experiments described in this manuscript. In fact, the use of a common software environment (and even the same script code) to perform simulations and conduct real-time experimentation eliminates the need for recruiting additional software to fulfil the task at hands, while avoiding the technical issues derived from converting different types of data representations. Moreover, the proposed framework allows potential users to have complete control for the processes involving the generation of the topological operators and implementation inside the global system of equations, therefore contributing to a better understanding of the physical phenomena and mathematics related to acoustic wave transmission and interaction with body tissues and implant materials. An advantage not shared by the commercial software codes, as the user, for the sake of convenience and faster computational processing times, can only actuate on the input parameters for the simulation model, including the location of the US source, material properties allocation and definition of material boundaries, regime of propagation, etc. Improvements to the framework proposed within the present manuscript can still be made in the future, concerning the aspects of increasing the domain resolution and processing speed by means of distributed computation in parallel, as well as the incorporation of simulations dealing with anisotropy analysis (tensor algebra) and the elastic/shear stresses posed by acoustic waves (or external loads) on body tissues and implant materials, as performed in typical Ultrasound Elastography imaging^33^. As stress fields may be different inside and locally around the solid implants, generation of multiple wave types can occur due to shear coupling that require, on one hand, more intense local mesh refinement in the interface implant-tissue to deal with the complex physical phenomena involved while, on the other hand, electronics must be prepared to handle different detection mechanisms and settings, including higher signal resolutions.

## Methods

### Full wave equation

**Figure 9:**
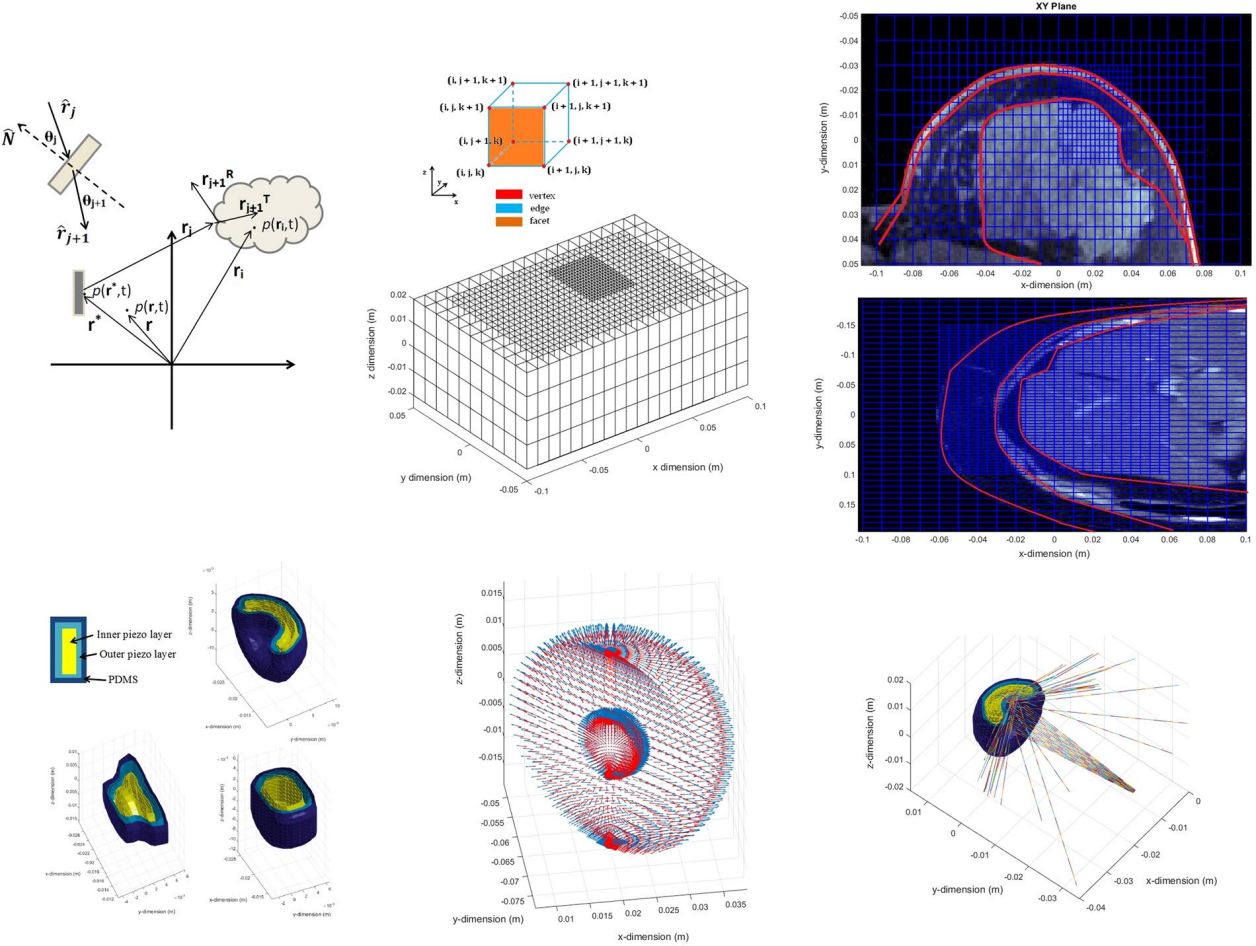
Considerations regarding the geometry of the 3D computational domain. (**a**) Generic representation of the structures present in a typical US imaging domain with positioning vectors. **r** is a generic point location in ${\mathbb{D}}$ experiencing a pressure *p*(**r**,*t*) whereas r* and **r**_*i*_ refer to points in the surface of the transducer ($\in$ ${\mathbb{S}}$) and inhomogeneity, respectively. **r**_*j*_ is an acoustic ray emanating from the source that will be deflected at the surface of the inhomogeneity, giving rise to reflected or transmitted rays, ${{\bf{r}}}_{j}^{R}$ and ${{\bf{r}}}_{j}^{T}$. (**b**) Multi-level resolution grid employed to cover the 3D domain of simulation with corresponding label scheme adopted to identify the vertices of each cubic element, with resolution equal to the length of the edge. (**c**) MRI transverse slices at the level of the breast (top) and liver (bottom) superimposed over the computational grid (in blue), with boundary contour lines (in red) highlighting relevant tissue discontinuities. (**d**) Geometrical configuration and layer composition for the implantable devices being tested: lens, cone and disk. (**e**) Surface normals oriented outward (blue) and inwardly (red) for the lens-shaped implant. Although the numerical system of US equations uses cubic elements in the algebraic matrices, for visualization purposes only, the elements defining borders are converted to curvilinear shapes by means of a tri-scattered data interpolation performed by MatLab. (**f**) Conceptual representation of the acoustic beam spreading effect and refraction produced by the implant.

In Ultrasound Imaging, the distribution of the acoustic pressure field at any point **r** in domain ${\mathbb{D}}$ results from a temporal and spatial convolution connecting the acoustic properties of the propagation medium with the geometry and excitation mode of the source transducer. In the other end, the signal collected by the detector (when different from the source) is typically modelled as a two-step mathematical convolution of the form^34^,

3 $$p({{\bf{r}}}^{\ast },t)={\tau }_{S}(t)\times {F}_{D}({\bf{r}})\times {H}_{SD}({\bf{r}},{{\bf{r}}}^{\ast },t)$$

where ${\tau }_{S}$ is the temporal electromechanical response of the transducer, *F*_*D*_ accounts for the density and propagation velocity variations produced by domain inhomogeneities and *H*_*SD*_ maps the time propagation delays due to refraction of the acoustic rays, from the source (represented by r*) to the spatial extent of the scattered field, as shown in Fig. 9a. Equation (3) has been solved for some transducer geometries and excitation regimes, when proper boundary conditions and structures in the propagation medium can be defined^35^. Writing the equation in its integral form, leads to an expression of the type,

4 $$p({{\bf{r}}}^{\ast },t)={\int }_{V}\mathop{\sum }\limits_{j}^{ray}\varUpsilon ({{\bf{r}}}_{j})\exp \left\{i\omega \left(t-\mathop{\sum }\limits_{i}^{loss}\frac{{{\bf{r}}}_{j}-{{\bf{r}}}_{i}}{{v}_{i}}\right)\right\}\exp \{-\mathop{\sum }\limits_{i}^{loss}{\mu }_{i}(\omega )[{{\bf{r}}}_{j}-{{\bf{r}}}_{i}]\}{T}_{S}(\omega )dV$$

with $\varUpsilon$ representing the nominal amplitude for the *j*^*th*^-ray originating at the source (and slit interfaces); the first and second exponential terms account for the frequency-dependent attenuation and phase-delay factors of the rays as they travel across the lossy medium. The attenuation coefficients ${\mu }_{i}$ represent inhomogeneities that dissipate some energy of the beam at the centre-frequency $\omega$ while producing phase delays induced by a shift in the propagation velocity denoted by *v*_*i*_. Within this hypothesis, different acoustic phenomena can be readily separated: refraction and scattering. While the former involves the evaluation of the acoustic rays as time-changing vectors in space, the latter is concerned with the calculation of all losses in the medium that are time-invariant. Hence, the solution for the full-wave equation is encountered in this paper by solving, independently, two Forward Problems of the form,

5 $$p({{\bf{r}}}^{\ast },t)={{{\mathscr{O}}}_{{\mathscr{P}}}}^{ray}({{\bf{r}}}^{\ast },{\bf{r}},t)\otimes {{{\mathscr{O}}}_{{\mathscr{P}}}}^{sct}({{\bf{r}}}^{\ast },{\bf{r}})$$

where ${{{\mathscr{O}}}_{{\mathscr{P}}}}^{ray}$ is the forward operator involved in ray-tracing calculation and ${{{\mathscr{O}}}_{{\mathscr{P}}}}^{sct}$ the full-domain scattering operator.

### Calculation of the scattering operator

The derivation of the pressure field equation in its differential form has been extensively reported in literature^34^. Neglecting second-order terms and assuming that the acoustic properties - velocity and density - only experience minor changes in the mean values, the equation can be stated as,

6 $${\nabla }^{2}p({\bf{r}},t)-\frac{1}{{v}_{0}^{2}}\frac{{\partial }^{2}p({\bf{r}},t)}{\partial {t}^{2}}=-\frac{2\Delta v}{{v}_{0}^{3}}\frac{{\partial }^{2}p({\bf{r}},t)}{\partial {t}^{2}}+\frac{1}{{\rho }_{0}}\nabla (\Delta \rho )\cdot \nabla p({\bf{r}},t)$$

where *p*(**r**, *t*) is the acoustic pressure measured at any point of the domain; *v*_0_ and $${\rho }_{0}$$ are the velocity and density of the background medium, respectively; $$\Delta $$*v* and $$\Delta \rho $$ are variations of the previous properties; and, finally, $${\nabla }^{2}$$, $$\nabla $$$$\cdot $$ and $$\nabla $$ are the Laplacian, divergence and gradient operators. The right hand side of the equation represents all wave interference phenomena that vanish in the absence of medium inhomogeneities, obtaining the homogeneous field generated by the source transducer or incident field. A common scheme adopted to further simplify the above equation is to take the Laplace domain in the limit *s* $$\to $$ $$-i\omega $$ so as to eliminate the temporal dependency of the pressure. The total pressure field is now solely given by a combination of the incident pressure field and the scattered field, presented in Eq. (7).

7 $${p}^{tot}({\bf{r}})={p}^{inc}({\bf{r}})+{p}^{sct}({\bf{r}})$$

By comparing the last two expressions, similarities are readily found which allow to re-write a simplified version of the equation in the integral form as^36,37^,

8 $${p}^{tot}({\bf{r}})={p}^{inc}({\bf{r}})+{\int }_{r{\prime} \in {\mathbb{D}}}{\mathscr{G}}({\bf{r}}-{\bf{r}}{\boldsymbol{{\prime} }})\Delta \gamma ({\bf{r}}{\boldsymbol{{\prime} }}){p}^{tot}({\bf{r}}{\boldsymbol{{\prime} }})dV+{\int }_{r{\prime} \in {\mathbb{D}}}{\mathscr{G}}({\bf{r}}-{\bf{r}}{\boldsymbol{{\prime} }})\{\nabla \cdot [\Delta \rho ({\bf{r}}{\boldsymbol{{\prime} }})\nabla {p}^{tot}({\bf{r}}{\boldsymbol{{\prime} }})]\}dV$$

with $\Delta \gamma$ being the medium contrast function that accounts for the differences in the complex propagation coefficient, ${\mathscr{G}}$ is the time-independent Green’s function and **r’** represents all the points in the domain ($\in {\mathbb{D}}$) other than the actual point **r**, this excluding the source transducer points (r* $\in {\mathbb{S}}$). The complex propagation coefficient $\gamma$ in this manuscript has followed a mathematical formulation of the type present in Eq. 9, in order to account for the power attenuation law found within biological tissues^1^.

9 $$\gamma ({\bf{r}})=\alpha ({\bf{r}})+i\beta ({\bf{r}})$$

The attenuation coefficient $\alpha$ and phase coefficient $\beta$ are derived themselves from a frequency-dependent model obtained from experimentation with biological tissue and described in literature (Table 1), replicated in here by Eq. 10.

10 $$\{\begin{array}{l}\alpha ({\bf{r}})=a({\bf{r}}\mathrm{)(2}\pi {)}^{-y({\bf{r}})}|\omega {|}^{y({\bf{r}})}\\ \beta ({\bf{r}})=\frac{\omega }{v({\bf{r}})}+a({\bf{r}}\mathrm{)(2}\pi {)}^{-y({\bf{r}})}tan[\frac{\pi }{2}y({\bf{r}})]\omega |\omega {|}^{y(r)-1}\end{array}$$

By its turn, the contrast functions for the density and propagation coefficient are calculated in accordance to Eq. 11, by taking into account the characteristics of the background medium (represented by ${\rho }_{0}$ and ${\gamma }_{0}$), while the mathematical formulation for Green’s function is provided in Eq. 12.

11 $$\{\begin{array}{l}\Delta \rho ({\bf{r}})=\frac{{\rho }_{0}-\rho ({\bf{r}})}{\rho (r)}\\ \Delta \gamma ({\bf{r}})={\gamma }_{0}^{2}-\frac{{\rho }_{0}}{\rho ({\bf{r}})}{\gamma }^{2}({\bf{r}})\end{array}$$

12 $${\mathscr{G}}({\bf{r}})=\frac{{{\rm{e}}{\rm{x}}{\rm{p}}}^{-{\gamma }_{0}({\bf{r}})}}{4\pi |{\bf{r}}|}$$

The incident field is obtaining by spatial convolution of the geometrical model for the source transducer with Green’s function for a background medium as Eq. 13 attests, completing ${{{\mathscr{O}}}_{{\mathscr{P}}}}^{sct}$.

13 $${p}^{inc}({\bf{r}})=i\omega {\rho }_{0}{\int }_{{{\bf{r}}}^{\ast }\in {\mathbb{S}}}{\mathscr{G}}({\bf{r}}-{{\bf{r}}}^{\ast })s({{\bf{r}}}^{\ast })dS$$

Finally, the resolution of the scattering operator is achieved using iterative methods that approximate the solution in terms of the total pressure in every step of the routine to the linearised system present in Eq. (14).

14 $${p}^{inc}({\bf{r}})={{{\mathscr{O}}}_{{\mathscr{P}}}}^{sct}[{p}^{iter}({\bf{r}})]$$

The Conjugate Gradient (CG) method is here adopted to provide an estimation to the scattered field with a standard routine procedure of the form,

15 $$\{\begin{array}{l}{{\bf{p}}}_{{\bf{0}}}={\bf{0}}\\ {{\bf{r}}}_{{\bf{n}}+{\bf{1}}}={{\bf{p}}}^{{\bf{i}}{\bf{n}}{\bf{c}}}-{{{\mathscr{O}}}_{{\mathscr{P}}}}^{sct}[{{\bf{p}}}_{{\bf{n}}}]\\ {{\bf{p}}}_{{\bf{n}}+{\bf{1}}}={{\bf{p}}}_{{\bf{n}}}+{\theta }_{{\bf{n}}}{\xi }_{{\bf{n}}}\\ error=\frac{||{{\bf{r}}}_{{\bf{n}}+{\bf{1}}}{||}_{2}}{||{{\bf{p}}}^{{\bf{i}}{\bf{n}}{\bf{c}}}{||}_{2}}\end{array}$$

where **p**_0_ is the initial estimate for the total pressure field (null vector), **p**_*n*+1_ is the updated pressure, ${\theta }_{n}$ the step size, ${\xi }_{n}$ the update direction and **r**_*n*+1_ the residual field that accounts for differences between the incident field and the estimated one. The step size and update direction are calculated by the system in Eq. 16, which involves the computation of the Hermitian adjoint for ${{{\mathscr{O}}}_{{\mathscr{P}}}}^{sct}$, represented in here by ${{\mathscr{O}}}_{{\mathscr{P}}}^{\ast \,sct}$.

16 $$\{\begin{array}{l}{\xi }_{-{\bf{1}}}={\bf{0}}\\ {\xi }_{{\bf{n}}}={{\mathscr{O}}}_{{\mathscr{P}}}^{\ast {\bf{s}}{\bf{c}}{\bf{t}}}({{\bf{r}}}_{{\bf{n}}})+\frac{||{{\mathscr{O}}}_{{\mathscr{P}}}^{\ast \,{\bf{s}}{\bf{c}}{\bf{t}}}({{\bf{r}}}_{{\bf{n}}}{)||}_{2}}{||{{\mathscr{O}}}_{{\mathscr{P}}}^{\ast \,{\bf{s}}{\bf{c}}{\bf{t}}}({{\bf{r}}}_{{\bf{n}}-1}{)||}_{2}}{\xi }_{{\bf{n}}{\boldsymbol{-}}1}\\ {\theta }_{{\bf{n}}}=\frac{||{{\mathscr{O}}}_{{\mathscr{P}}}^{\ast \,{\bf{s}}{\bf{c}}{\bf{t}}}({{\bf{r}}}_{{\bf{n}}}{)||}_{2}}{||{{{\mathscr{O}}}_{{\mathscr{P}}}}^{{\bf{s}}{\bf{c}}{\bf{t}}}({\xi }_{{\bf{n}}}{)||}_{2}}\end{array}$$

### Calculation of the ray-tracing operator

The ray-tracing method is proposed in this manuscript to solve the wave equation, based on straight ray propagation and refraction, whenever it occurs on the domain interfaces. Since the field of a ray propagating in the same medium is an undisturbed spherical wave, when reaching an interface with different refraction index, the ray will be distorted into a different direction through the discontinuity (ray transmission) or, eventually, reflected back as stated in Eq. (17).

17 $$\varUpsilon ({{\bf{r}}}_{j})={\varUpsilon }^{T}({{\bf{r}}}_{j+1})+{\varUpsilon }^{R}({{\bf{r}}}_{j+1})$$

The amplitude of the waves at each side of the discontinuity is obtained by Snell’s Law as,

18 $${n}_{j}\,{\rm{s}}{\rm{i}}{\rm{n}}({\theta }_{j})={n}_{j+1}{\rm{s}}{\rm{i}}{\rm{n}}({\theta }_{j+1})$$

where ${n}_{j}$ and ${n}_{j+1}$ are the refraction indexes of the *j*^*th*^ and *(j*+*1)*^*th*^ mediums, respectively, whereas ${\theta }_{j}$ and ${\theta }_{j+1}$ are the angle of incidence and refraction. The direction of the ray emerging from the interface relative to the incident one is computed by Eq. (19) with the *a priori* knowledge of the normal vector to the interface, $\hat{N}$, and the direction of incidence, ${\hat{r}}_{j}$.

19 $${\hat{r}}_{j+1}=\frac{{n}_{j}}{{n}_{j+1}}[\hat{N}\times (-\hat{N}\times {\hat{r}}_{j})]-\hat{N}\sqrt{1-{\left(\frac{{n}_{j}}{{n}_{j+1}}\right)}^{2}(\hat{N}\times {\hat{r}}_{j})\cdot (\hat{N}\times {\hat{r}}_{j})}$$

The inclusion of the corresponding phase-delay for each ray in the domain of simulation concludes the computation of ${{{\mathscr{O}}}_{{\mathscr{P}}}}^{ray}$, either the ray being tracked is a newly-created ray at the source, a vanishing one or simply a reflected/refracted version of a previous ray in time. The full expression for the forward operator involved in ray-tracing can now be represented in accordance to Eq. (20).

20 $${{{\mathscr{O}}}_{{\mathscr{P}}}}^{ray}=\sum _{j}\varUpsilon ({{\bf{r}}}_{j})\exp \left\{i{\omega }_{0}t-\mathop{\sum }\limits_{i}^{loss}\frac{{{\bf{r}}}_{j}-{{\bf{r}}}_{i}}{{v}_{i}}\right\}$$

### Computational mesh

The domain of simulation is discretized into orthogonal cell-complexes whose vertices, edges and facets are associated with an initial orientation, as depicted in Fig. 9b. Each complex is a tensor product in Cartesian coordinates of the form ${V}_{i,j,k}:=[{x}_{i},{x}_{i+1}]\times [{y}_{j},{y}_{j+1}]\times {[{z}_{k},{z}_{k+1}]}_{i\mathrm{=1,...,}I-\mathrm{1;}j\mathrm{=1,...,}J-\mathrm{1;}k\mathrm{=1,...,}K-1}$, where the vertices (${x}_{i},{y}_{i},{z}_{i}$) are the coordinates along the *x-*, *y-* and *z-*directions, respectively, with a total number of cells given by ${N}_{C}=(I-\mathrm{1)}\cdot (J-\mathrm{1)}\cdot (K-\mathrm{1)}$. The stacking of the cells generates the computational grid with resolution given by the length *h* of the edge and pressure value assigned to the cell barycenter. Since, large-scale 3D domains have a large discrepancy in length that compromise computational performance, the strategy adopted here to surpass this setback consists in designing a local mesh refinement over punctual domain regions where a small-scale detail is relevant. This subgridding scheme must prevent a difference greater than one in the level of resolution between adjacent neighbours and the co-existence of elements with different resolutions puts into jeopardy the one-to-one topological correspondence found in the differential operators related to ${{{\mathscr{O}}}_{{\mathscr{P}}}}^{sct}$. Now, the matrix entries for the operators cease to have a discrete coefficient set $\in \{-\mathrm{1,0,1\}}$, as a maximum number of four different neighbours becomes possible for a coarser element in contact with a finer grid, instead of just one; on the other side, elements with higher resolution can only have a maximum of one neighbour, either at the same level or with a lower resolution element in any direction. The tracking of the intricate relation of every element with the direct neighbours is performed at the same time as the refinement process takes place, in the form of a sparse matrix of the type,

21 $${N}_{\{component\}\{orientation\}\{direction\}}$$

where $\{component\}$ refers to any of the axis component of the quantity in study; $\{orientation\}$ varies according the *x-*, *y-* or *z-*orientation of the neighbour; and $\{direction\}$ is the positive (+) or negative (–) direction of orientation. Each sparse matrix has dimensions of ${{\mathbb{R}}}^{{N}_{C}\times {N}_{C}}$, with row index *l* identifying the element and column *m* its neighbours. The entry ($l,m$) thus contains the value one if the neighbour exists in the same resolution level and if not, a set of interpolation weights (summing-up to one) are redistributed along the entries containing the position of the neighbours. Within this terminology, the discrete differential operators are given as,

22 $${\rm{div}}=3D-{N}_{xx+}-{N}_{yy+}-{N}_{zz+}$$

23 $$\{\begin{array}{c}{{\rm{grad}}}_{{\rm{x}}}=D-{N}_{xx-}\\ {{\rm{grad}}}_{{\rm{y}}}=D-{N}_{yy-}\\ {{\rm{grad}}}_{{\rm{z}}}=D-{N}_{zz-}\end{array}$$

where $D$ $\in$ ${{\mathbb{R}}}^{{N}_{C}\times {N}_{C}}$ is a diagonal matrix with non-null entries set to one. The remaining physical quantities, namely pressure field and contrast functions, follow a similar lexicographical ordering of the elements, with diagonal matrices of the form ${{\mathbb{C}}}^{{N}_{C}\times {N}_{C}}$, ${{\mathbb{R}}}^{{N}_{C}\times {N}_{C}}$ and ${{\mathbb{C}}}^{{N}_{C}\times {N}_{C}}$, respectively.

### Phantom segmentation

The mathematical relations just described are implemented using Matlab for 3D anatomical phantoms obtaining from MRI scans freely available online from the **Cancer Imaging Archive** as high-resolution DICOM images, down-converted to 256x256 JPEG format. The segmentation of the tissues inside each 2D slice was performed based on the grey level attributed by the imaging modality to each individual point, with clusters of similar *pixels* being identified as a whole tissue. Additional image filtering by means of a Gaussian mask with *5-by-5* pixels is also employed to smooth-out sharp transitions and eliminate isolated points within the anatomical slices. The acoustic properties - density, velocity and attenuation - assigned for the most common biological tissues are displayed in Table 1, together with the materials employed in the design of the implantable device. The stacking of different 2D transverse slices creates the 3D phantom whose contrast properties are allocated into complex diagonal matrices of the form ${{\mathbb{C}}}^{{N}_{C}\times {N}_{C}}$.

The identification of the interfaces between tissues is performed once the density value for each *voxel* is set. Beginning at the geometric centre of a particular tissue (or cluster), line segments spread radially in all directions until the end segment reach a different density value at the border. By connecting together all these boundary points, a contour line is drawn around each relevant tissue involved in the computation of ${{{\mathscr{O}}}_{{\mathscr{P}}}}^{sct}$, as depicted in Fig. 9c. For the scattering operator, the incident pressure field is calculated prior to the GC routine by discretizing the source transducer over the 3D domain grid by applying Eq. 13. Since only planar surface transducers are employed, the number of source points in the grid is given by the area occupied by the surface with radius set to 5 mm. Three different levels of resolution are assigned to the main computational grid, with $h$ values set to 10 mm, 5 mm and 2.5 mm. Higher resolution elements cover the space connecting the transducer to the implantable device, whereas the coarser ones sustain the limits of the domain. The remaining elements with *h* = 5 mm cover the majority of the biological tissues, yielding ${N}_{C}$ equal to 12752 and 28072 for the breast and abdomen phantoms, respectively.

The spatial resolution for the ray-tracing operator is dictated by the wavelength of the acoustic rays. The rays originating at the source are allowed to propagate for distances up to a prescribed number of wavelengths, with a limit set to 1000. Variations in layer thickness, composition and geometrical arrangement of the implant will then selectively transmit, block or deflect the trajectory of the rays and promote phase-delays for those rays (eventually) reaching back the transducer. In the computational simulations the aperture diameter for the detector is allowed to change for the discrete range $\in$ [0.01, 0.07] m in 0.01 m steps.

### Implantable device design

The implant was modelled as a true solid and composed by two innermost layers with different piezoelectric materials (Table 1), which were then covered by an external layer made of bio-compatible PDMS whose acoustic impedance is similar to fat tissue, as shown in Fig. 9d. The computation of the normal to the implant surface $\hat{N}$ for each layer is of paramount importance in order to obey Eq. 19. Both the outward and inwardly-oriented surface normals are computed during the design of the implantable device, which will intersect the wave-front of the acoustic beam when emanating from the exterior or interior of each layer, respectively, as depicted in Fig. 9e. These normals are given by the radial component to the surface by the time the different layers of the implant are being created, for the linear and curvilinear geometries. For the tissues, the interfaces are not so regular and the normals are provided by the normal component of the last line segment spreading radially from the centre of the tissue to the boundary. Since one cannot infinitesimally discretise each surface, the intersection error in terms of the distance between the geometrical points defining $\hat{N}$ and $\hat{r}$ is within a sphere of radius set to half the distance obtained by $\mathrm{4500/}frequency$, with 4500 m.s^−1^ representing the average propagation velocity for tissues and piezos employed in the paper. The double piezoelectric layer can have one of six different compositions for the inner/outermost pair - **PZN-PT/LiNbO**_3_, **PZN-PT/BaTiO**_3_, **PZN-PT/PVDF**, **PZT-5A/LiNbO**_3_, **PZT-5A/BaTiO**_3_ and **PZT-5A/PVDF** - and thickness is in the discrete set of **0.5 mm/1 mm**, **1 mm/2 mm**, **1.5 mm/3 mm**, **2 mm/4 mm** and **2.5 mm/5 mm**.

### Electronic circuit for experimental validation

**Figure 10:**
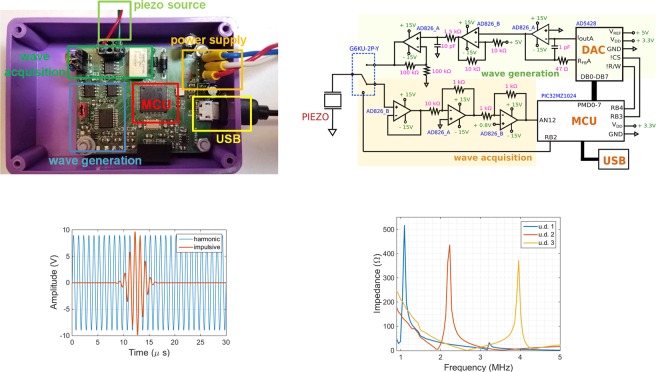
External electronic device designed to generate ultrasonic acoustic waves in the harmonic/impulsive regimes of stimulation and detect the returning echoes. (**a**) Simplified electronic schematic of the main components and signal connections composing the different modules of the external US device. (**b**) Printed circuit board with main connections to the source transducer (piezo), external power supplies and computer (USB). (**c**) Waveforms produced by electronics to stimulate the source transducer (1 MHz frequency). (**d**) Different impedance curves obtained for the US transducers used in the experimentation (u.d. 1, u.d. 2 and u.d. 3), as measured by a commercial impedance analyzer.

The generation of the acoustic stimulation signal and detection of the echoes originated at the implantable device are performed by the customized electronic board shown in Fig. 10a, designed around an high performance micro-controller (MCU, PIC32MZ1024, Microchip, Chandler, AZ, USA) commanding the modules for **wave generation** and **wave acquisition**, respectively. For the generation of the stimulation signal, two regimes of wave propagation were considered: harmonic (sinewave) and impulsive (Gaussian pulse). To that end, two waveform templates were stored inside the program memory of the MCU, representing one period of a sinewave (20 data points) and a Gaussian pulse (100 data points). According to the selection of ultrasonic transmission ($\in$1, 10 MHz), these data points are released from the micro-controller at precise temporal instants to an external digital-to-analogue converter (DAC, AD5428, Analog Devices, Norwood, MA, USA) with parallel interface and 8-bit of resolution, as shown in Fig. 10b. Bipolar range of the stimulation signal required two external amplifiers (AD826, Analog Devices) to convert the positive unipolar samples present at the output of the DAC to their bipolar equivalent. Additional 1^*st*^-order low-pass filtering of the signal with cut-off frequency around 11 MHz is performed by a passive RC network, before amplification by a factor of 2 V/V in a non-inverting amplifier configuration (AD826, Analog Devices) in order to set the amplitude range of the stimulation signal to the interval $\in$ [−10, 10] V. The signal is then applied to one of the input terminals of the piezo transducer, acting as the US source, by converting the voltage signal to an equivalent acoustic pressure wave. Figure 10c depicts some examples of waveforms applied to the transducer, which is composed by PVDF material sandwiched by two silver electrodes (diameter of 2.5 cm). The selected transducers employed for experimentation (P727, Precision Acoustic Ltd., Dorset, UK) can have different resonant frequencies according to the thickness of the transducer - 1 mm, 0.5 mm and 0.25 mm - and set for operation in the range from 1 MHz to 4 MHz. Figure 10d shows the impedance amplitude for the different transducers (u.d. 1, u.d. 2 and u.d. 3) as measured by a commercial impedance analyzer (model E4990A, Agilent Technologies, Santa Clara, CA, USA).

For the detection of the returning echoes, the input terminal of the source transducer is re-directed by a relay (G6KU-2P-Y, Omron, Tokyo, Japan) to an operational amplifier (AD826, Analog Devices), as shown in Fig. 10b (wave acquisition), followed by signal amplitude attenuation with a factor of 10 V/V through a reduction amplifier topology (AD826, Analog Devices), before DC baseline level shift at 1.6 V imposed by an unitary-gain inverting amplifier (AD826, Analog Devices) in order to confine the amplitude of the detected echo signals to the interval range $\in$ [0, 3.3] V, as imposed by the internal analogue-to-digital converter (ADC) of the MCU. Digitization is performed at a rate of 20 million samples *per* second (SPS) during a 100 *μ*s time interval with a resolution of 10-bit (or equivalently, 3 mV) and the digitized samples are transferred afterwards to a computer by Universal Serial Bus (USB) communication at a speed of 1 Mbps.

## Data Availability

All the computational code was developed in Matlab (R2018b) and it can be found through the link: https://figshare.com/s/456f76500bf9f1d3235c. A description of every function and script is also provided. The code involved in the creation of the computational mesh, implementation of the mathematical formulas and design of the phantoms/implants was entirely developed from scratch, with only the functions for visualization in Matlab being recruited.
